# Rapid nondestructive measurement of bacterial cultures with 3D interferometric imaging

**DOI:** 10.1038/s41598-019-43839-7

**Published:** 2019-05-30

**Authors:** Curtis Larimer, Michelle R. Brann, Joshua D. Powell, Matthew J. Marshall, Jonathan D. Suter, R. Shane Addleman

**Affiliations:** Pacific Northwest National Laboratory, Richland, WA USA

**Keywords:** 3-D reconstruction, Optical imaging, Microbiology techniques, Biofilms, Interference microscopy

## Abstract

The agar culture plate has played a crucial role in bacteriology since the origins of the discipline and is a staple bioanalytical method for efforts ranging from research to standard clinical diagnostic tests. However, plating, inoculating, and waiting for microbes to develop colonies that are visible is time-consuming. In this work, we demonstrate white-light interferometry (WLI) as a practical tool for accelerated and improved measurement of bacterial cultures. High resolution WLI surface profile imaging was used for nondestructive characterization and counting of bacterial colonies on agar before they became visible to the naked eye. The three-dimensional (3D) morphology of Gram-negative (*Pseudomonas fluorescens*) and Gram-positive (*Bacillus thuringiensis*) bacterial species were monitored with WLI over time by collecting surface profiles of colonies on agar plates with high vertical resolution (3–5 nanometers) and large field of view (3–5 mm). This unique combination of sensitive vertical resolution and large field of view uniquely provided by WLI enables measurement of colony morphologies and nondestructive monitoring of hundreds of microcolonies. Individual bacteria were imaged within the first few hours after plating and colonies were accurately counted with results comparing favorably to counts made by traditional methods that require much longer wait times. Nondestructive imaging was used to track single cells multiplying into small colonies and the volume changes over time in these colonies were used to measure their growth rates. Based on the results herein, bioimaging with WLI was demonstrated as a novel rapid bacterial culture assay with several advantageous capabilities. Fast nondestructive counting of colony-forming units in a culture and simultaneous measurement of bacterial growth rates and colony morphology with this method may be beneficial in research and clinical applications where current methods are either too slow or are destructive.

## Introduction

Light wave interference—the process in which two waves can add together constructively or destructively cancel each other out when they overlap—and the patterns it creates has been foundational to scientific research since Isaac Newton first studied ring patterns in optical glasses in 1717. Albert Michelson and Edward Morley used a light interferometer to disprove the theory of “luminiferous aether” in 1887, and more recently, the Laser Interferometer Gravitational-Wave Observatory (LIGO) used two large optical interferometers to detect gravitational waves and validate Albert Einstein’s century old theories^1^. Optical interferometers have found broad application in many fields of basic and applied science because they can be used to make sensitive distance measurements. Uniquely, optical interferometers are capable of measuring sub-nanometer height variations over large areas because interference fringes can be observed for all pixels in an image simultaneously. A common industrial application of optical interferometry is the precision measurement of surface topography and roughness^2^. As an example, light interferometry was used to verify nanometer scale smoothness of the 1.3 m diameter mirror segments that make up the primary mirror of the James Webb Space Telescope^3^. In this work, we explore the use of a sensitive interferometric microscopy technique, white-light interferometry (WLI), to observe the small changes in the topology of bacterial microcolonies as they grow on agar plates.

Optical interference has been employed extensively for biology and medicine^4^ in the form of phase contrast and differential interference contrast (DIC) microscopy^5^. In recent years, optical coherence tomography (OCT) has also gained wide use in ophthalmology where it is used to visualize the internal microstructure of eye tissues^6^. Though WLI operates on the same principle as OCT, it has not garnered wide use in biological imaging. Both OCT and WLI are forms of low coherence interferometry: OCT employs a near-infrared laser while WLI uses a lower wavelength broadband (“white”) light that is typically provided by a light emitting diode source. WLI has higher spatial and axial resolution than OCT and because it is a surface imaging technique it cannot resolve continuously through the depth of a sample in the way that OCT can. WLI is optimized for industrial applications with stable hard reflective surfaces and as a result, dynamic wet samples that are typical of *in vitro* imaging in microbiology are challenging.

In our prior work we described techniques to utilize the unique capabilities of WLI to image the surface topology of hydrated bacterial biofilms^7–10^. Other uses of WLI for bioimaging have focused on exploiting the difference in optical path length through cells^11^. Herein, we explored the use of WLI to image bacterial colonies grown on agar plates. Some of the most common means for qualitative and quantitative analysis of bacteria involve culturing organisms in petri dishes that contain growth nutrients and agar. The growth of a single bacterium in a defined location in the agar plate can give rise to a proliferating colony that is typically visible within one to three days, but incubation times can easily extend to weeks depending on the species and growth conditions. In biomedical and food safety settings, quantifying the colony-forming units (CFU) on agar plates is used to determine levels of contamination. Agar plates can also be selective and/or differential through the addition of specific nutrients, indicators, antibiotics, or salts (e.g., Mannitol for *S. aureus* detection), and therefore can also be used as a diagnostic tool for assessing antibiotic resistance or the presence of hallmark biochemical features indicative of a pathogenic organism.

In many cases standard culture-based counting methods do not support precise, reproducible counts of mixed cultures, especially when there is a mixture of viable and viable but non-culturable bacteria^12^. Many imaging techniques have been used to aid and accelerate colony counting. Fluorescence microscopy is notable for its use in many culture-dependent and culture-independent methods for bacterial enumeration. Additionally, fluorescent biomolecular stains and labels identify presence of nuclear acid, cellular integrity, and other features^12^. Nanoparticles are also commonly used to label bacteria for counting and identification,^13^ but nanoparticles, biomolecular stains, and high energy light needed to excite them can have toxic effects on bacteria^14,15^. As such, standard optical imaging is preferred for computerized imaging systems and software that automate plate counting^16,17^. While these software tools speed up the counting process they still require incubation and growth until colonies become visible and they are not currently able to collect other information about colony morphology or growth rate.

In light of recent advances in WLI instrumentation and advances in automated analysis of 3D images, we endeavored to determine if WLI could be used to speed enumeration of bacterial colonies on agar by observing them before they can be seen with traditional methods. We aimed to nondestructively measure surface changes caused by individual bacteria and microcolonies using WLI in two configurations: a combination of high magnification lenses (100x) to achieve fine lateral resolution and low magnification lenses (1.375x) to image the largest possible field of view. Experiments were designed take advantage of the nondestructive nature of WLI imaging to observe bacterial colonies as they grew over time in order to measure changes in colony morphology and growth rate. The Gram-negative bacterium *Pseudomonas fluorescens* (PF) and the Gram-positive bacterium *Bacillus thuringiensis* (BT) were used for these studies because both are model organisms that share some of the microbiological characteristics of more pathogenic bacteria. PF is often used to model biofilm formation and as a surrogate for *Pseudomonas aeruginosa*, a pathogen that is associated with cystic fibrosis, pneumonia, and sepsis^18^. BT, in addition to its use as an environmentally friendly insecticide, is used as bench-safe model for studying certain attributes of its pathogenic relatives, *B. anthracis* and *B. cereus*^19^. The results described below indicate that WLI is capable of accurate enumeration of Colony-Forming Units (CFU) counts from agar plates while also enabling accurate quantification of growth rates and other morphological attributes.

## Results and Discussion

### WLI imaging of agar culture plates

A WLI microscope is similar to an upright brightfield optical microscope. The objective lenses have either Michelson-type or Mirau-type interferometers which split illumination to a reference arm and the sample. Interference is observed at the image sensor when recombined light from the reference and sample surfaces has travelled equal optical distance (see Supplemental Video 1 for an animated illustration of a Mirau-type WLI system). WLI imaging results in a 3D profile of the surface with 3–5 nm resolution in the Z-direction (resolution in the X-Y plane is diffraction limited as in traditional optical microscopy). This very fine vertical sensitivity enables resolution of fine details that are not otherwise visible. An image takes about 30–60 seconds to acquire. Commercially poured plates were used throughout this study, except where specifically noted, because the plates were consistently smoother (lower than 50 nm root mean squared surface roughness as measured by WLI) than plates poured in our laboratory.

**Figure 1:**
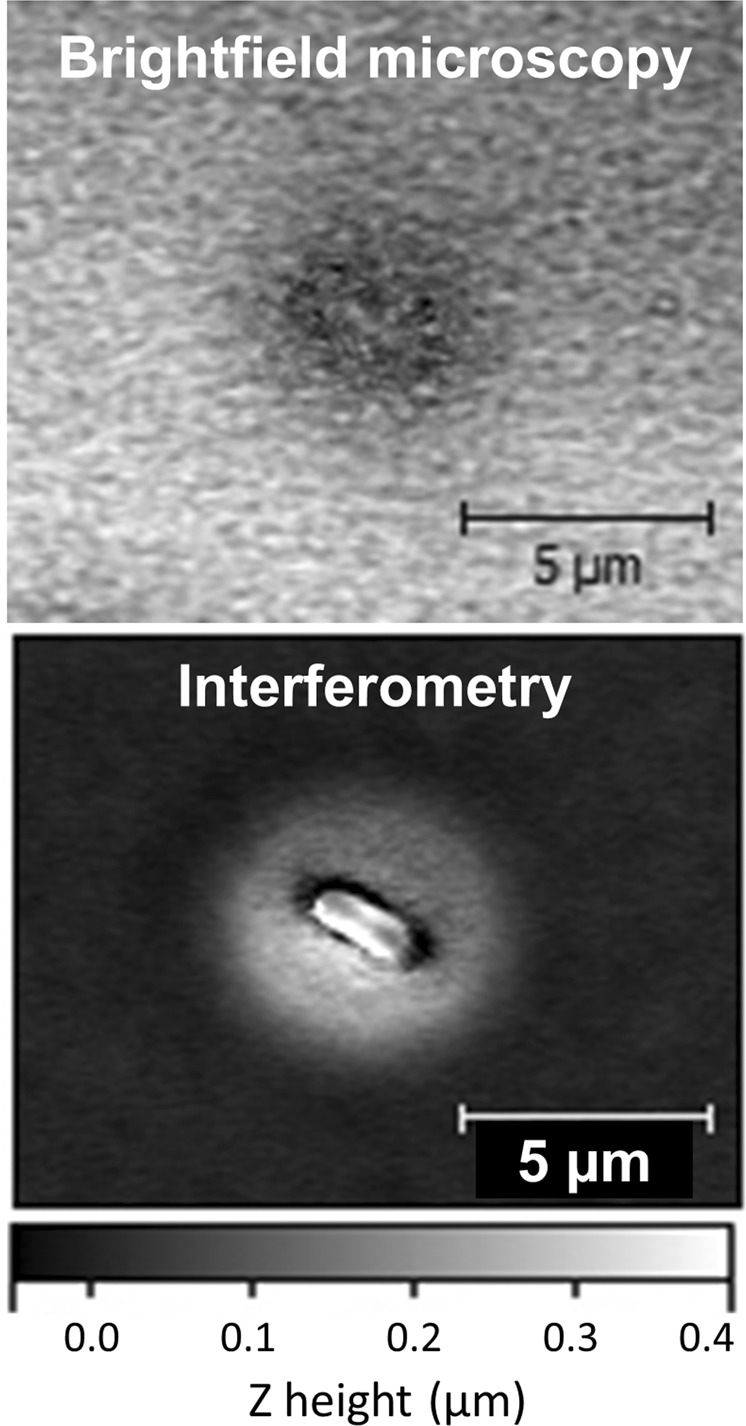
A comparison of a standard brightfield optical microscope image (top) with an interferometric microscopy image (bottom) of a single cell of *Pseudomonas fluorescens*. The interferometric image resolves z-height variations of less than 0.4 microns which increases contrast over the standard optical image. The interferometric image is falsely shaded by z-height according to the grayscale bar shown below the image. Images were collected with a 50x objective lens (NA = 0.55) and 2x field of view lens. Magnification is indicated by scale bars.

Figure 1 shows traditional 2D optical and 3D interferometric images of a sample of PF on the surface of an agar plate, illustrating a key advantage of WLI: i.e., that 3D imaging with fine vertical resolution also improves optical contrast in the X-Y plane as compared to a bright field image. Bacterial cells are weakly absorbing, so they exhibit very little contrast in bright field images. Just as in fluorescence imaging, the improved contrast of interferometric imaging makes it easier to resolve the shape and size of small microbes on agar plates at the single cell level. The volume of a single microbe may be as small as ~1 femtoliter (10^−15^ l or 1 µm^3^) but the theoretical volumetric resolution of WLI (5 nm height change from a single pixel while using a 100x magnification lens configuration) is ~50 zeptoliter (5 × 10^−20^ l). As a result, there is high confidence that WLI can resolve volume changes that result from cell division and multiplication.

**Figure 2:**
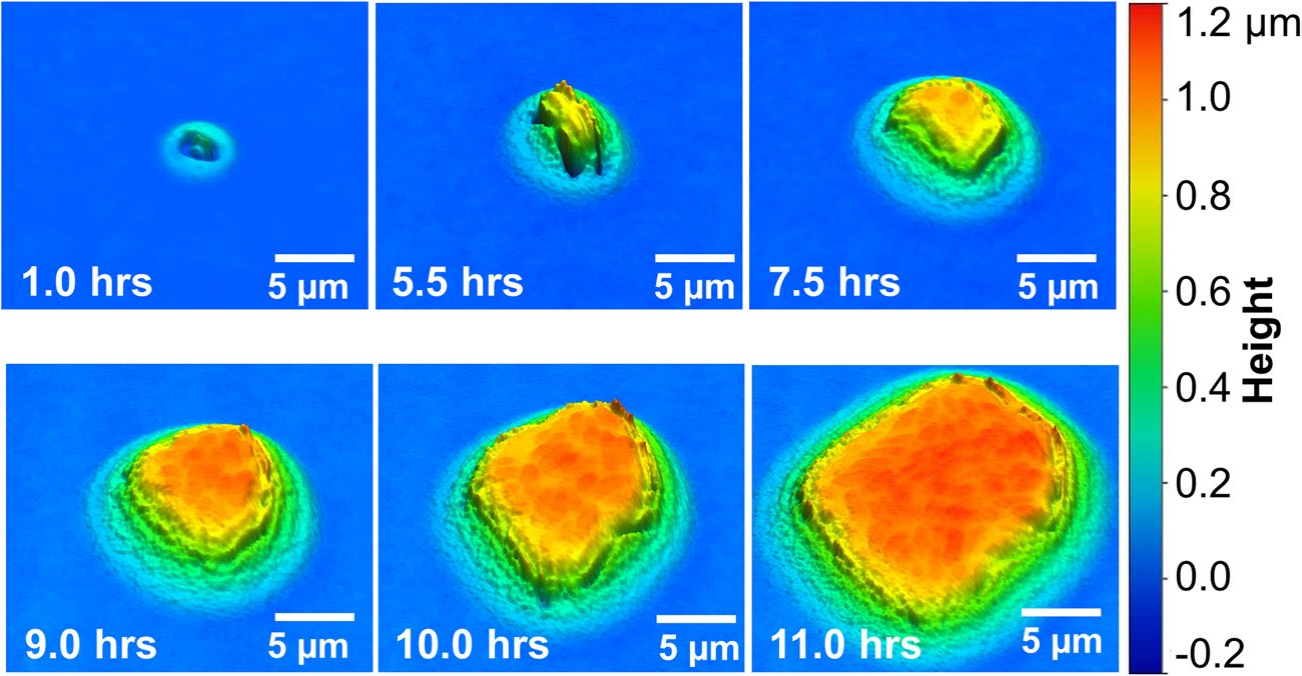
A series of WLI surface images of a single PF colony on an agar plate show growth from a single cell 1 hr after plating to a multi-cellular microcolony at 11 hrs. Fine morphological features on the surface of the colony appear to reveal outlines of individual bacteria. The horizontal scale is shown with a bar in each image. The vertical scale is represented by false color as shown at right. Images were collected with a 50x objective lens (NA = 0.55) and 2x field of view lens.

Figure 2 shows the continued sequence of high resolution 3D optical profile images of the PF sample shown in Fig. 1 captured as the single microbe grew into a microcolony. In the early hours (approximately 1–6 hrs), it was possible to observe individual cells multiplying. This is more clearly observed in Supplemental Video 2, which is a time series of all of the recorded images. At the 9, 10, and 11 hour time points shown in Fig. 2 the surface of the colony appears to have rod-like features that resemble individual microbes. The colony appears to grow uniformly in all directions, which is consistent with the phenotype of PF plate colonies that are known to grow into a rounded ball-like shape after about 24 hours^20^. Fig. 2 shows that WLI enables nondestructive imaging of the surface morphology of early-stage microcolony growth. It should be emphasized that because of the varying heights of features across numerous samples, it was not possible or reasonable to use a single uniform color scale for all WLI images in this manuscript. Instead, we have chosen a color scale, height range, and lateral scale that accurately highlights and accentuates the unique features in each set of images.

**Figure 3:**
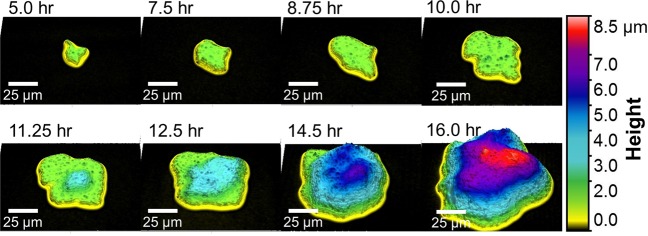
A series of WLI surface images of a single BT colony on an agar plate show growth of a small irregularly shaped colony 5 hr after plating. The microcolony grew laterally in the first 10 hrs before a second bacterial layer emerged at 11.25 hrs. The height of each layer is ~2 µm (similar to the expected diameter of BT cells). The lateral scale is shown with a bar in each image. The vertical scale is represented by false color as shown at right. Images were collected with a 50x objective lens (NA = 0.55) and 2x field of view lens. Magnification is indicated by scale bars.

Figure 3 shows a series of high resolution WLI images of BT grown on an agar plate that was prepared and poured in our laboratory. A different morphology was observed for this species compared to PF. The BT microcolony seen at 5 hrs is irregularly shaped and appears to grow laterally in a single layer (green) for more than 10 hours before a second layer of microbes (light blue) begins to emerge. Each layer has a height of approximately 2 µm. A third layer of microbes (dark blue) is visible at 14.5 hours. This series of morphological changes is also shown in Supplemental Video 3.

### Quantified growth measurements

**Figure 4:**
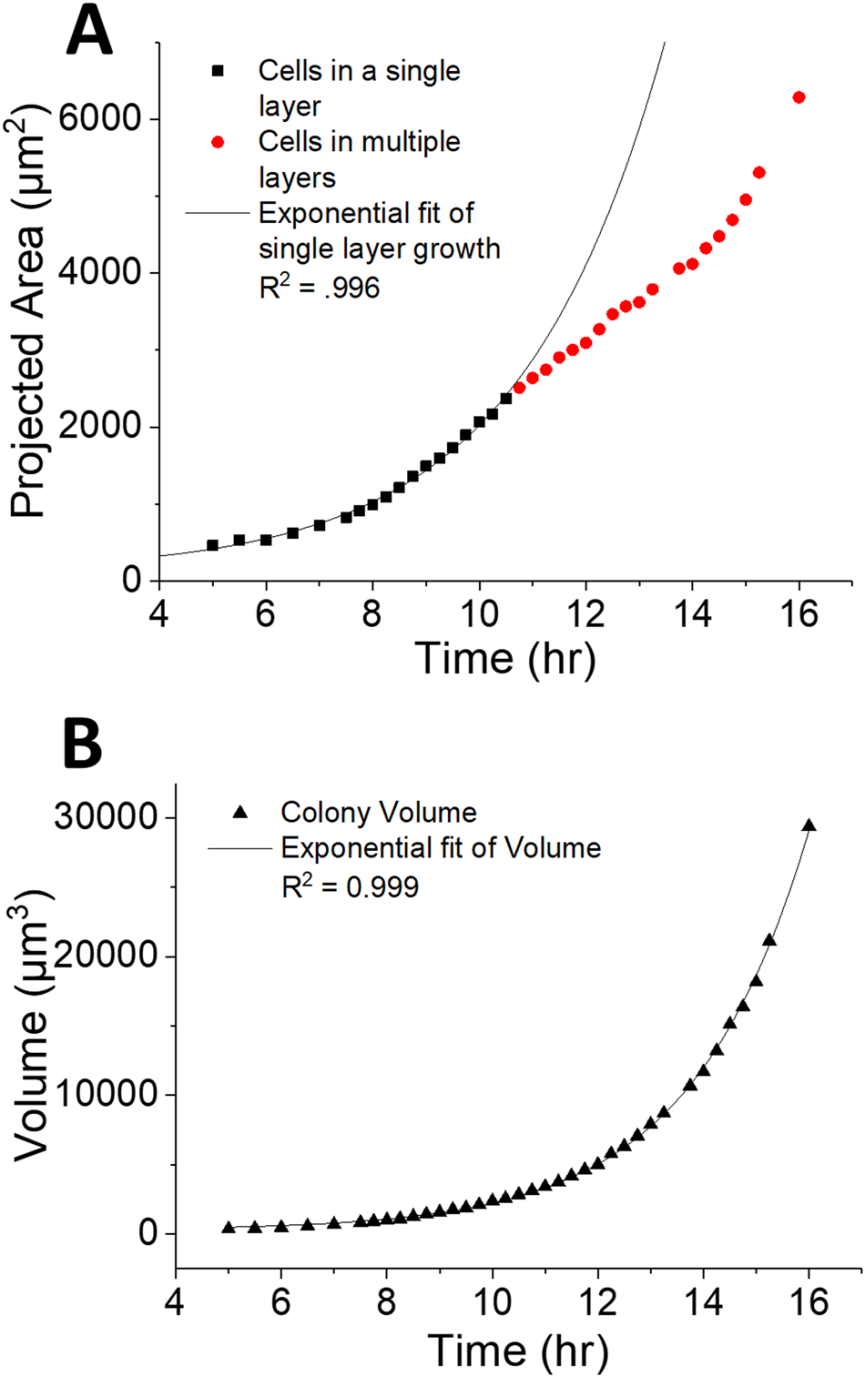
Growth of a single BT colony as measured by WLI (select images shown in Fig. 3). The projected area (**A**) of the colony grew exponential while the colony maintained a single layer (▪) but then did not follow the exponential fit curve after the colony developed multiple bacterial layers () emerged at 10.5 hrs. In contrast, the volume (**B**) (▲) of the colony grew exponentially throughout the experiment regardless of colony morphology.

The image series in Figs 2 and 3 enabled observations of morphological changes in the early stages of colony formation and measurement of growth without disrupting or interrupting growth of the colony. This raised the intriguing possibility that the image data could also be used to quantify growth rate by observing changes in the volume of a colony over time. Figure 4 is a comparison of the calculated volume of a single BT colony to its projected area (A) as measured from the images shown in Fig. 3 (and Supplemental Video 3). Data were collected from 37 WLI surface profile images of a single bacterial colony. Volume and projected area were calculated by first determining the outline of the colony. Projected area was simply calculated from the number of pixels contained within the colony’s outline. Volume was calculated by multiplying the area of each pixel by its Z-height and summing over the whole colony. Figure 4A shows that the colony grows laterally in the early hours (up to 11 hours) and this growth is also observed in the expansion of projected XY area. The projected area follows an exponential growth trend, and as a result projected area appears to be an accurate measure of growth during this period. After 11 hours, the colony continues to grow laterally, but the projected area no longer follows an exponential growth curve because a second layer of microbes emerges at this time. After 11 hrs a portion of the colony’s growth is vertical so total growth can no longer be measured as an increase in XY area alone. By comparison, Fig. 4B shows that the volume of the colony grows exponentially throughout the experiment, even when multiple layers of cells are seen in the images. In this case, measurement of volume, as enabled by WLI, imaging resulted in more accurate tracking of colony growth through the duration of the experiment.

Fitting the volume data with an equation for exponential growth resulted in a growth constant (*k*) of 0.45 ± 0.004 hr^−1^ and a doubling time (*T*) of 1.54 ± 0.01 hrs, which is in line with literature values^21^. The fitting function allows us to project future growth as well as estimate volume of the colony prior to the start of imaging. For example, the volume of the “colony” at the time of plating—a time at which we assume there is a single BT cell—was estimated to be 21.6 ± 1.4 µm^3^. If we assume this is the volume of each cell we can estimate that there are ~17 cells in the colony at 5 hrs. Given the doubling rate of 1.54 hrs we would expect there to be approximately 9–10 cells 5 hrs after seeding a colony with a single cell so we can assume that the bacteria grew more quickly in the first few hours of incubation.

### Monitoring multiple bacterial colonies

A limitation of the data shown in Figs 3 and 4 is that it only shows or derives from a single colony imaged with high resolution. The small field of view that results from using high magnification lenses captured detailed images of a single colony, but did not allow for observation and analysis of other colonies. As described above, WLI has the unique ability to record images with high axial resolution (~3 nm) even at low magnification (e.g., a 2.5x magnification lens). To demonstrate and evaluate this capability, BT and PF cultures were also imaged with WLI using the widest possible field of view. A diluted culture was pipetted onto the agar plates using a small volume (2 µl) in order to make a spot on the plate that could be imaged in its entirety in one field of view. This volume of bacterial culture created ~5 mm diameter discs akin to miniature replicates of full-scale agar plates (typically 100 mm diameter) with each disc containing ~10–100 individually separated cells. Under normal circumstances it would not be possible to enumerate colony forming units in a 5 mm disc because the colonies would grow too large and merge together before they became visible. But with WLI microscopy it is possible to do so by collecting 3D surface images containing all of the colonies.

**Figure 5:**
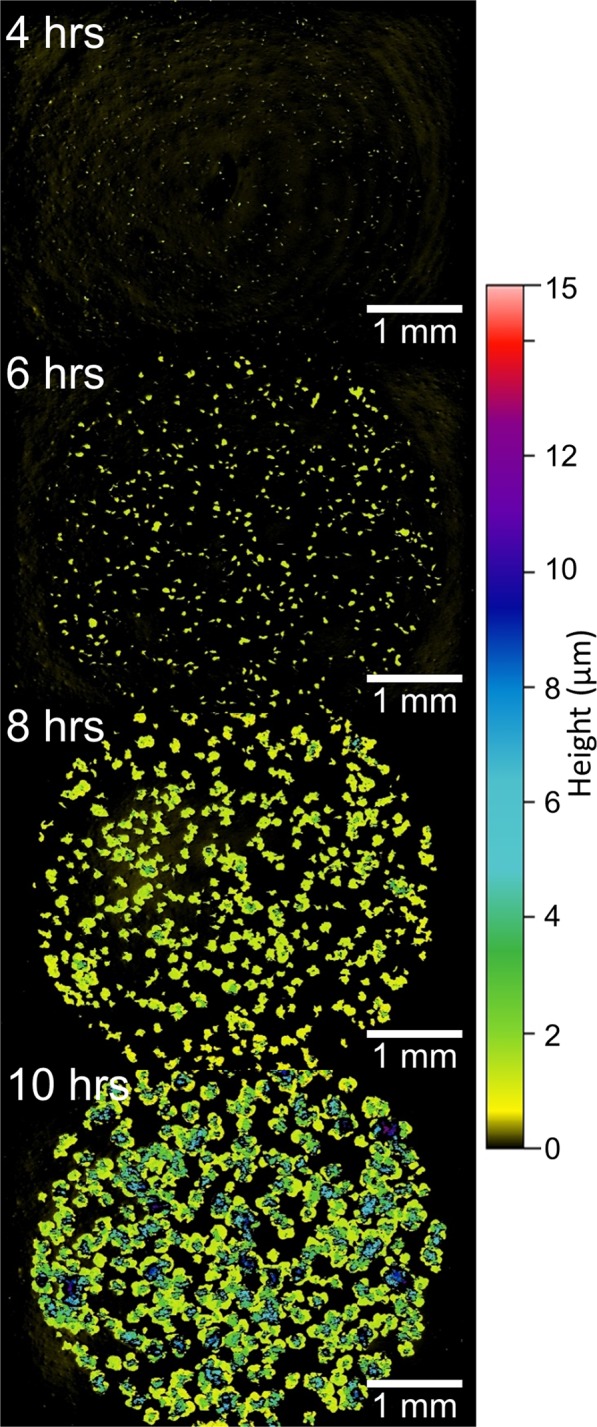
A series of surface profile images of 2 µl spots of diluted (10^−3^) culture of BT show the growth of many microcolonies over time. Imaging a wide field of view enables analysis and enumeration of many bacterial colonies at once. Lateral scale is shown by the bar in each image. Vertical scale is represented by false color as shown at right. Images were collected with a 2.5x objective lens (NA = 0.07) and 0.55x field of view lens.

**Figure 6:**
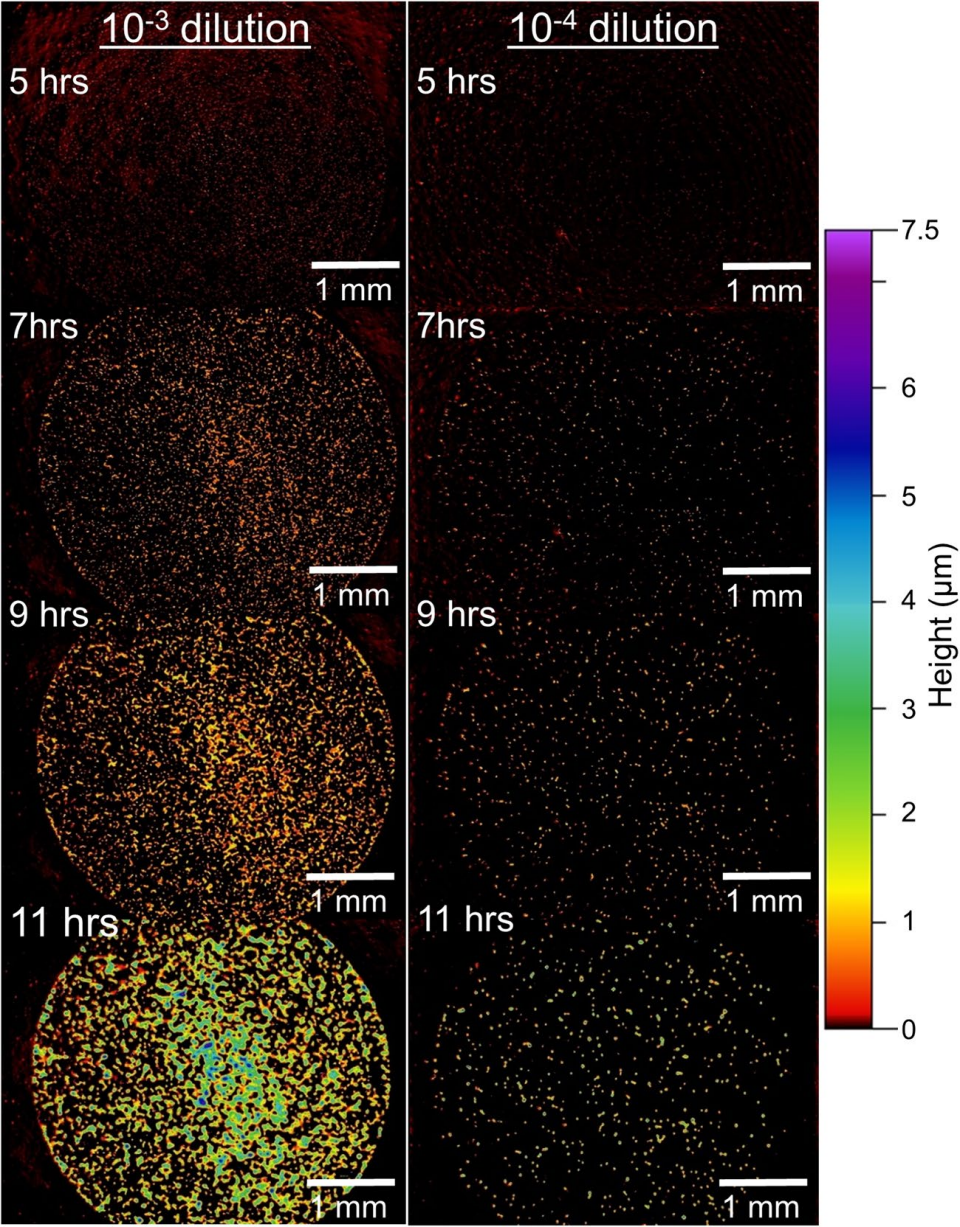
A series of surface profile images of 2 µl spots of diluted culture of PF show the growth of many microcolonies over time. The culture imaged on the left was diluted by three orders of magnitude (10^−3^) while the same culture diluted by four orders of magnitude (10^−4^) is shown on the right. The images show hundreds of microcolonies growing and eventually merging together. Lateral scale is shown by the bar in each image. Vertical scale is represented by false color as shown at right. Images were collected with a 2.5x objective lens (NA = 0.07) and 0.55x field of view lens.

Figures 5 and 6 show a selection of images taken over time of plated spots of BT and PF, respectively. The full series of images for both species are shown in Supplemental Videos 4 and 5. The images in Figs 5 and 6 show a constellation of hundreds of microcolonies as they grow and eventually merge together. In Fig. 5, the characteristic irregular shapes of BT colonies (plated as a 10^−3^ serial dilution) are clearly visible. Also, emergence of multiple layers are visible in the image series, just as they were in the high resolution images of a single colony.

Figure 6 includes images of PF at two dilutions (10^−3^ and 10^−4^). In the 10^−3^ dilution the colonies are densely packed and are clearly seen merging together at 11 hrs. In the more dilute culture, bacterial colonies are not clearly visible until 7 or 9 hrs, because they were smaller and appeared to grow more slowly.

The growth of BT and PF colonies in Figs 5 and 6 was further analyzed by counting the colonies in each image and measuring the volume of each colony. An analysis algorithm was written to automate the search for colonies in each image series and calculation of their morphological characteristics (e.g., projected area, volume, height, etc.). This was done because it was often difficult to manually count small colonies in the images—the small protrusions simply blend in with background textures. For example, with visual inspection of the WLI data one cannot see colonies in images of the 10^−4^ dilution until about 9 hrs after the plating. Using the automated algorithm, colonies were identified and counted in the earliest image, just 2 hrs after placing the culture spot on the plate. The analysis algorithm searched for peak regions above the background by calculating surface statistics from multiple “islands” of data in each sample. Filters were applied to remove data that was incorrectly labeled (e.g., single pixel peaks or excessively large regions). It is likely that some non-colonies are identified at the earliest times (i.e., because contaminants or protrusions in the growth medium could be morphologically similar to very small colonies). However, non-colonies would not grow over time so it becomes much easier to filter them out at later measurement times by simply excluding smaller islands. At the conclusion of the experiment, samples were collected from merged colonies and streaked on agar to confirm that the bacteria were viable (i.e., that the imaging did not kill the colonies).

The algorithm counted 87.3 ± 5.5 colonies in the images of PF cultures in the 10^−4^ dilution (averaged from 4 images of different culture spots). Analysis of the 10^−3^ dilution (performed at some earlier times when the colonies were well separated) detected upwards of 800 colonies in a single image. Just as in standard plate counting, the WLI method can be used with diluted cultures (10^−5^ or more) to ensure that there are fewer colonies in a single image and that they are more spread out, thus facilitating an accurate count.

**Figure 7:**
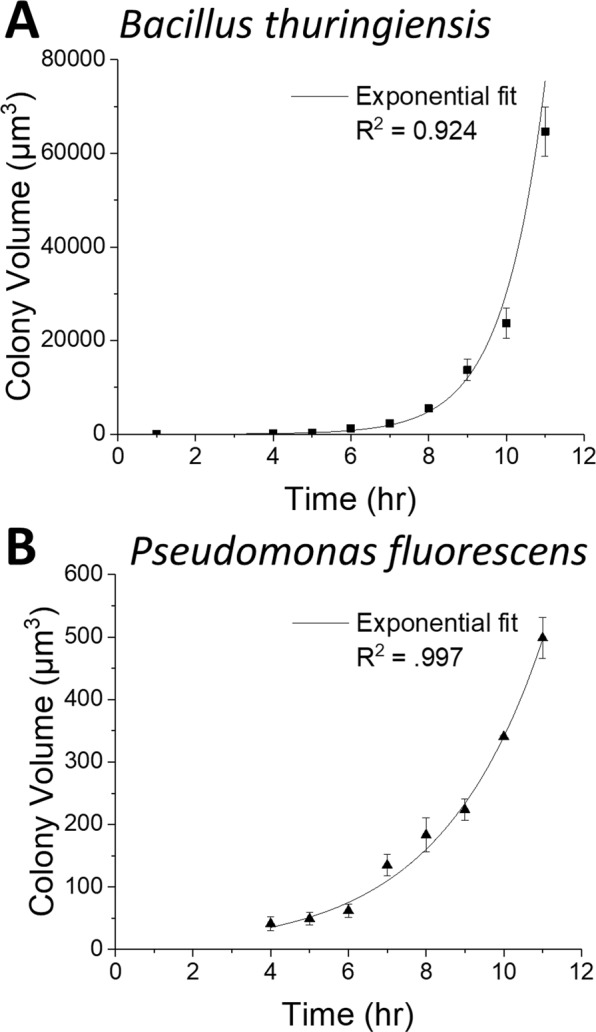
The calculated volume of multiple microcolonies of BT (**A**) and PF (**B**) are plotted as a function of time. Each species follows an exponential growth curve as shown by the overlaid fit lines. The *Bacillus* colonies grew more quickly than did the *Pseudomonas* colonies. Error bars show standard deviation from ~60–80 microcolonies shown in each wide-field-of-view image.

Images of BT cultures also contained between 50 and 750 separate microcolonies, depending on the dilution of the culture prior to plating. As in the high resolution images, calculated volume of both PF and BT cultures showed exponential growth, as shown in Fig. 7. As described above, the growing volume of the colonies was used to calculate the growth rate. In this experiment the doubling times were 0.75 ± 0.04 and 1.84 ± 0.28 for BT and PF respectively. The growth rate for BT in this experiment is different from the rate measured above because it was cultured on a commercially-available plate rather than a homemade plate to facilitate imaging a smooth background over a large area. BT grew faster and larger than PF over the duration of the 11 hr experiment. These analyses confirm that it is possible to measure the morphology of many hundreds of microcolonies in parallel with WLI microscopy. Further, the tracking the volume of colonies over time can be used to calculate growth rates.

### Comparison of CFU counting by WLI and traditional methods

**Figure 8:**
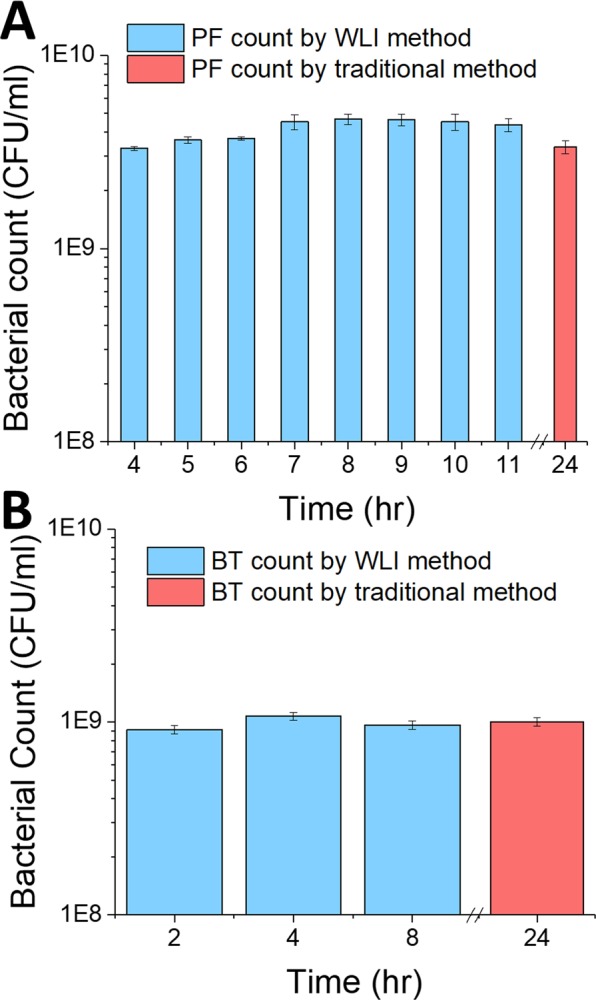
Comparison of bacterial counts in agar plate cultures using WLI (blue) and traditional method (red) for PF (**A**) and BT (**B**). The graphs show that the WLI method is accurate and has comparable error range to the traditional method. Error bars show standard deviation (*n* = 4).

To compare results of enumeration by WLI and traditional plate counting, we set up dilution series of cultures of both BT and PF and plated dilutions on 10-cm agar plates for standard plate counting at the same time as we plated 2 µl samples for enumeration by WLI. We expected CFU counts to remain consistent over time because while the bacteria are multiplying exponentially, the number of colonies on the plate is not changing. Figure 8A shows that there was a small increase in CFU counts of PF from 4 to 8 hours. It was clear during the analysis that in the first few hours after plating it was more difficult to distinguish PF bacteria from the background because the colonies did not protrude far enough above the background to be accurately separated by our algorithm. Thus, some shallow colonies were not counted. With time all of the colonies grew taller and were easier to detect, resulting in the small increase in counts. It also became easier to apply filters to remove any errant counts. The number of colonies counted declined after 8 hrs because some colonies grew large enough that they overlapped and were undercounted as a result. Figure 8 shows that both methods are in close agreement. Determination of CFUs by WLI at the earliest time were substantially similar to the count made by a standard manual plate counting method 24 hours later.

The CFU count of the PF culture at 4 hrs using the WLI method (3.3e9 ± 8.2e7 CFU/ml) was very similar to the count made at 24 hrs (3.4e9 ± 2.5e8 CFU/ml) using the traditional method. Likewise, the CFU count of the BT culture at 8 hrs using the WLI method (9.63e8 ± 1.42e8 CFU/ml) was not significantly different from the count made at 24 hrs (1.00e9 ± 1.29e8 CFU/ml) using the traditional method. The uncertainty of the WLI method is nearly the same as the traditional colony counting method.

These results indicate that WLI can be used to enumerate CFU in plate culture with several advantages over traditional plate counts: CFU can be measured much earlier than with traditional methods—perhaps as early as 2 hrs. The method uses fewer and smaller plates than standard methods, and the culture may be plated in fewer dilutions, reducing a time-consuming step. Thus, replacing manual plate counts with enumeration by WLI results in an order of magnitude decrease in time needed to measure CFU counts and perhaps a 100x reduction in materials.

### Observation of changes in colony morphology

**Figure 9:**
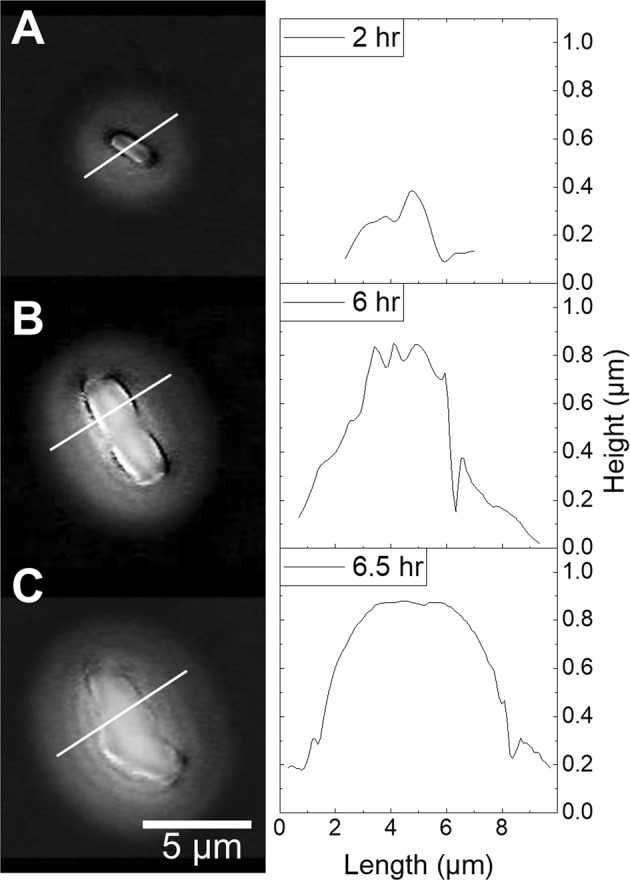
A series of WLI images taken with high magnification shows (**A**) a single cell soon after plating. The bacteria multiplied and formed a small colony as seen in (**B**). WLI generate cross-sectional profiles at right distinctly show one protrusion at 2 hrs in (**A**) and three side-by-side protrusions at 6 hrs (**B**) with another three cells visible at the ends. Just 30 minutes after the image shown in B, the morphology of the microcolony changed and was noticeably smoother, a possible indication of EPS formation. Lateral scale is shown by the bar in each image. Vertical scale is represented by grayscale false color with a 0–1 µm vertical range. Images were collected with a 50x objective lens (NA = 0.55) and 2x field of view lens.

As shown above, WLI can be used to observe both the morphology of growing bacterial colonies and volumetric data can be used to calculate their growth rates. Images were further analyzed to determine if other morphological changes could be identified. Figure 9 shows three images of a growing PF colony. The initial image clearly shows a single PF cell. Over time the cell divides and new cells are visible, though somewhat less clearly. Extracting cross-sectional line profiles from the WLI data of the colony (shown to the right of each image) assists in delineating individual bacteria. For example, Fig. 9B shows the colony 6 hrs after plating. The cross-sectional profile shows three clear protrusions at the surface of the colony, which indicates three separate cells. There appear to be three more cells adjacent to the ends of these cells. The volume of the colony at 6 hrs is 22.03 µm^3^, which is approximate 6 times larger than the initial single cell volume. Noticeably, the surface morphology of the colony changes dramatically between 6 hrs and 6.5 hrs. The undulations of individual bacteria transformed into a more uniform mass. We hypothesize that this morphological change is an indication that the small colony has begun producing or secreting extracellular polymeric substance (EPS), a characteristic trait of biofilm formation. If confirmed, this preliminary result would suggest that WLI can resolve this transition with high spatial and temporal precision. Future research will be required to better understand if the morphology changes observed correspond with phenotypic change from a planktonic to a biofilm state. It would be of particular interest given that this method is nondestructive and does not require the use of the use of biomolecular staining or contrast agents.

### Imaging mixed cultures

**Figure 10:**
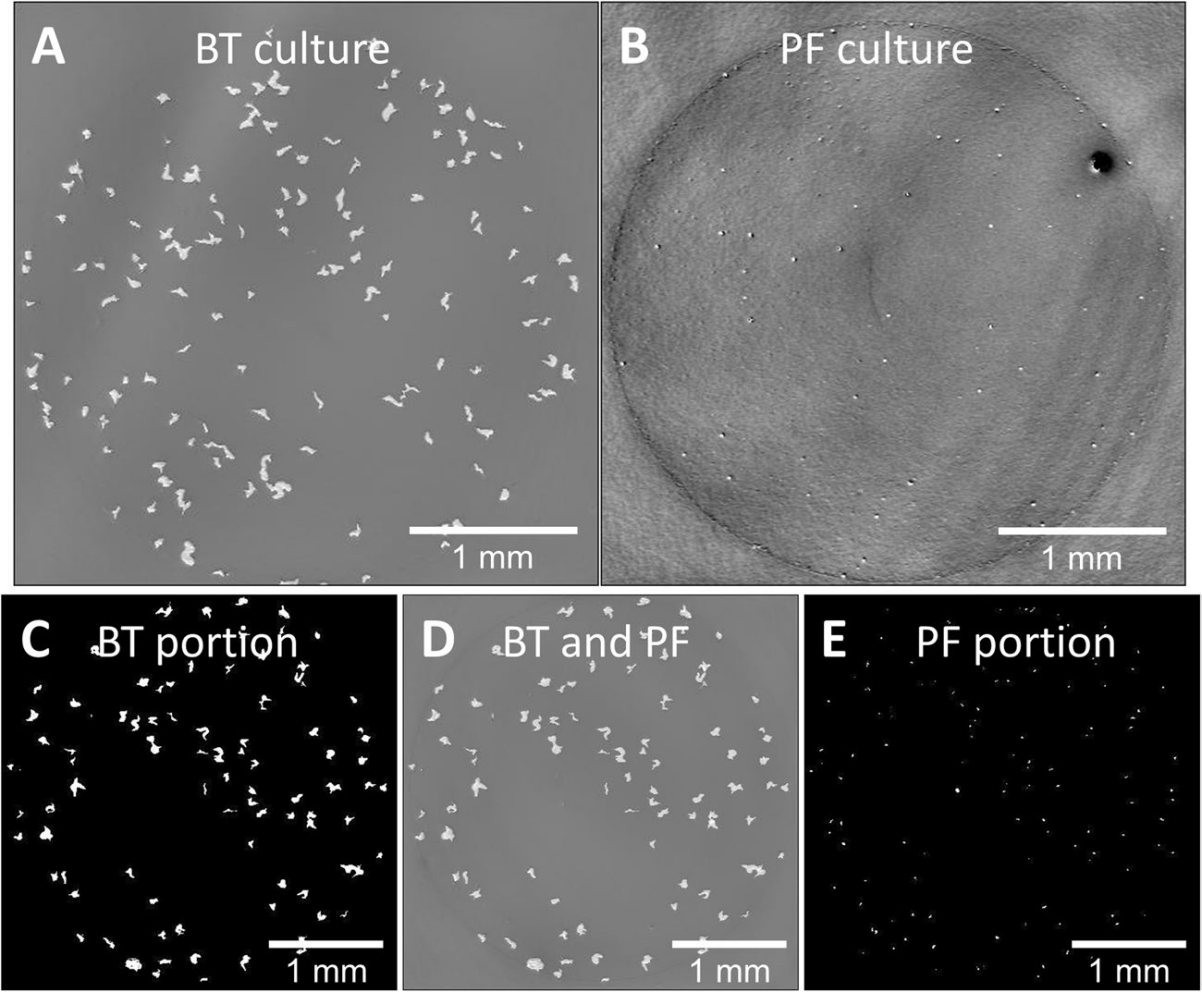
WLI surface profiles of agar plates with (**A**) BT, (**B**) PF and (**D**) a roughly 50/50 mixture of both. The two species in the mixed culture shown in (**D**) were identified by morphological traits (colony height and volume) and separated into two separate images by threshold filtering. (**C**) shows the BT portion of the culture and (**E**) shows the PF portion. Lateral scale as shown by the bar in each image. Vertical scale is represented by grayscale false color with a −1.8 to 1.8 µm vertical range for (**A,D**). (**B**) Has a −150 to 150 nm vertical range. (**C**) Is colored via threshold for objects taller than 1 µm and (**E**) is colored via threshold between 80 and 180 nm. Images were collected with a 2.5x objective lens (NA = 0.07) and 0.55x field of view lens.

The results above show that WLI has many potential applications in the study of monospecific bacterial samples. However, biofilms and clinical and environmental samples are almost always mixed cultures. An experiment was conducted to determine if WLI could be used to differentiate species in a simple mixed culture. Three plates were prepared with cultures of BT, PF, and a mixture of both. Images were collected 2, 4, and 8 hrs after plating. The composition of the culture on each plate (i.e., the proportion of each species) was easily identified at 2 hrs despite being unknown prior to imaging. Figure 10 shows images of the plates at the 8 hr mark. There is a clear morphological difference between BT and PF because BT forms larger and more irregularly shaped colonies while PF formed smaller rounded colonies. It is straightforward to separate the BT and PF portions of the mixed culture by filtering morphological traits measured with WLI (colony height and volume). Further work is required to extend this technique to mixed cultures with more constituent species and with more similar colony morphologies.

### Imaging colonies in a sealed system

**Figure 11:**
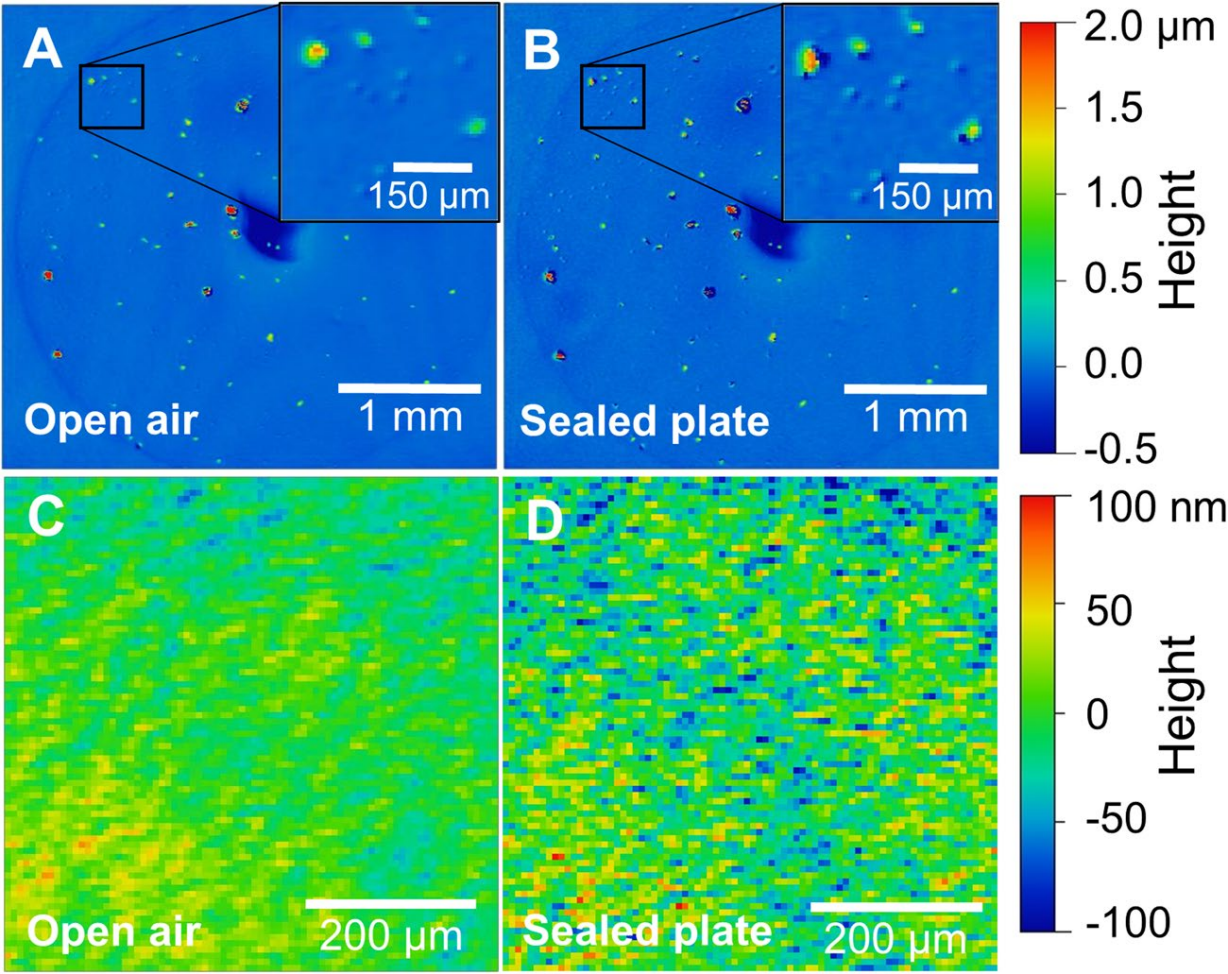
Comparison of WLI surface profile images of PF culture captured in two states: (**A**) in open air and (**B**) in a system sealed with a plate cover. Covering the plate resulted in a small increase in background noise. Inset images show strong correlation of fine morphological details. Comparison of smooth region of agar as imaged through open air (**C**) and through the glass lid of a sealed plate (**D**). A small increase in texture of the plate is lower than the size of bacterial microcolonies and does not significantly hinder accurate counting of colonies in profile images. Lateral scale as shown by the bar in each image. Vertical scale is represented by false color as shown at right. Images were collected with a 2.5x objective lens (NA = 0.07) and 0.55x field of view lens.

Another capability of WLI is that one can image a sample through transparent media. Imaging closed plate cultures would reduce the risk of contamination, especially for slow growing bacteria and species that require strict biosafety controls, and would allow for greater control of aerobic/anaerobic conditions and environmental conditions such as humidity. A final experiment was conducted to determine if it was possible to enumerate bacterial colonies on an agar plate that was closed with a lid and sealed. Figure 11 shows a comparison of a plate culture imaged in open air and the same culture imaged in a sealed plate.

Most of the experiments in this study were performed with open agar plates. Exposure to dry laboratory air caused the agar to shrink over time. Especially when imaging at high resolution, this was evident because the colony of interest continuously lowered from its original position and slowly shifted laterally as well. This made it difficult to maintain the culture in focus. A method for imaging with a closed plate was developed in part to mitigate this problem; however, if left in place the interior of the lid was prone to accumulate condensation that inhibited imaging. Moreover, a standard polycarbonate plate lid could not be used without optical compensation in the reference arm of the interferometer. In the end, an optically compatible lid was made for closed plate imaging. A plate cover was modified to remove a section of polycarbonate and replace it with a thin coverglass window. The thin coverglass was smoother, more even, and caused minimal change to the optical path length between the objective lens and the sample.

There is a modest increase in image noise between Fig. 11A,B, which show the same PF culture in an open plate and a sealed plate, respectively. Several of the colonies in Fig. 11B have dark outlines that are not present in Fig. 11A and this gives the impression of higher contrast. However, it should be noted that WLI cannot always definitively identify a surface for every pixel in an image and as a result there is often a small percentage of pixels that do not have height data. In this figure, pixels without height data are reproduced in black. WLI imaging often cannot determine heights on sharply inclined surfaces such as the edges of a protruding colony and this limitation is exacerbated when a cover glass is placed in the optical path.

The magnified inset images illustrate that differences caused by imaging through a transparent plate cover are not large enough to impact the ability to accurately count CFUs in the culture. The average number of colonies in the culture as measured with an open plate was 50 ± 3.6 (averaged from the image seen in Fig. 11A and two others). The colonies were counted again with the plate closed with a total of 51.7 ± 5.8. This closed plate imaging was possible without a compensated objective because the depth of field of the low magnification lens (2.5x) was large enough to accommodate the change in focal position caused by the cover glass^7^. A higher magnification lens would require compensation.

Figure 11C,D are magnified views of the data in 11A and 11B, respectively. They show the same smooth region of the agar plate with no microcolonies. Here an increase in image noise when viewing through the lid of the sealed plate was observed as an increase in surface roughness (S_q_) 18.3 nm without the lid to 28.9 nm with it. In future work, it may also be suitable to use a compensated objective^22^ to correct for optical effects introduced by a modified or unmodified plate lid. This demonstration of CFU enumeration in a sealed system shows that WLI could be integrated into existing standard procedures without introducing risks of contamination. The ability to ascertain CFU counts shortly after plating also means that data can be collected before a fast-growing contaminant overtakes colonies of interest.

### Benefits and limitations of WLI imaging

The purpose of this report was to describe the capabilities, benefits, and limitations of WLI imaging as an alternative or supplement to traditional bacterial colony counting on agar plates. These tasks are fundamental to many microbiology studies and clinical tests. A unique feature of WLI is the ability to collect high resolution vertical information even with a relatively large field of view. This enables a snapshot overview of a miniature sized plate culture that is akin to traditional plate reading practices. The results of the studies presented here show that WLI can be faster than traditional methods for colony counting. Furthermore, the enumeration of liquid culture spots as little as 2 µl in volume on traditional agar plates affords significant multiplexing as many dilutions can be placed on a single plate therefore saving time, materials, and labor costs. WLI can also be used with high magnification lenses to nondestructively image a single microbe as it grows into a microcolony. As a result, WLI provides detailed information about the morphology and growth rate of a colony and may indicate phenotypic changes such as secretion of EPS and transition to a biofilm state. In particular, the volumetric information that can be gleaned from 3D data results in more accurate tracking of growth rate than projected area could. Importantly, this information regarding growth rate can also be collected with low magnification lenses that result in a wide field of view that contains hundreds of microcolonies. The ability to view and enumerate bacteria in closed plates may be especially useful for slow growing species that are prone to being overgrown by contaminants. Additionally, the closed plate environment may be necessary for pathogenic agents that require handling under strict biosafety controls. In future work, we aim to develop methods to track growth of individual microbes in parallel rather than in aggregate.

Limitations of this WLI approach include the requirement for very smooth agar plates. Although we found commercial plates to be adequate for our measurements, overall, current plates are not optimized for the WLI imaging process. Plates prepared from powdered agar were typically not smooth enough and presented challenges in imaging. Commercially poured agar plates were surprisingly smooth but still not optimized for this application. The sensitivity of the WLI method presented here could improve with plates optimized for surface smoothness. Even with commercial plates, great care was taken to avoid touching the surface of the agar with the plastic disposable pipette tips when transferring small culture volumes because the tip easily left an indentation in the agar (see the dark depression at the center of Fig. 11A). Use of robotic pipetting would avoid this issue. It was also found, for example, that the plate edge often interfered with the movement of the objective lenses at different magnifications. In the future, it may be desirable to design an agar plate to best mitigate WLI’s limitations while making best use of advantages like the need for substantially smaller plate area.

WLI instrumentation has not been traditionally used in microbiology laboratories nor for microbiological applications, and features of the instrumentation could be improved for these uses. Certain aspects of WLI, such as finding focus and leveling the sample are more involved than with standard optical microscopy^23,24^. It should be possible to employ and improve automated instrument controls that are used in mechanized quality checks for some industrial processes^2^ to simplify instrument use. The resulting surface profile images may also include imaging artifacts such as the batwing or interferometric slope effects^25^. While many of these effects can be corrected with appropriate filtering, over processing the data can also lead to filtering artefacts (such as vignetting) in the images and to misinterpretation of the data.

A final limitation is in the procedures for quantitative analysis. For this study the analysis and enumeration of microcolonies was largely conducted manually because programmed algorithms do not exist for some tasks. In the future it would be desirable to integrate and automate the sampling, imaging, and analysis steps. This would be especially useful to transition WLI imaging to greater use in microbiology research or clinical practice.

## Conclusions

White-light interferometry is a sensitive optical imaging technique that has not been used extensively in microbiology. Results of this study support the conclusion that WLI enables nondestructive live cell imaging of bacteria growing on agar plates. One can observe individual cells without the need for staining to provide contrast and view a time series on the same colony or set of colonies during their growth. Data acquisition is relatively fast (~30 seconds per image) and can produce a high-resolution 3D surface profile across a range of magnifications. The experiments performed in this work revealed several advantageous findings: WLI is capable of nondestructive direct monitoring of growth rate (single and multi-colony), rapid direct colony counting (many colonies in the same field of view), and high-resolution imaging of surface morphology. Future research will be required to determine if the methods presented here can detect phenotypic changes such as EPS production. Results show that WLI can be used to count CFUs within 2–4 hrs of plating and the results are comparable to counts made by traditional methods 24 hrs after plating. Further research could develop the methods presented here into rapid high-throughput culture assays.

## Methods

### WLI instrument and software

In this work, two imaging configurations were used: one to achieve high lateral resolution and the other to image a wide field of view. WLI surface profiles were measured by a Bruker Counter Elite GT-I white-light interferometer operated in vertical scanning mode. All “high resolution” images (Figs 1–3 and 9 and Supplemental Videos 2 and 3) were collected using a 50x objective (NA 0.55) with 2.0x field of view lens, resulting in spatial sampling of 0.1 µm. Likewise, all “low resolution” or “wide field of view” images (Figs 5, 6, 10 and 11 and Supplemental Videos 4 and 5) were collected using 2.5x objective (NA 0.07) with 0.55x field of view lens resulting in a spatial sampling of 7.2 µm. Imaging areas were 4.6 × 3.4 mm for the wide field of view configuration and 62 × 47 µm for the high-resolution configuration. A green LED was used for illumination in all cases in order to maximize horizontal resolution. The noise threshold was set to the minimum value, 0.001. No compensation was used when imaging through thin glass coverslips. As discussed in detail in our previous work, it was found that the low magnification lens could tolerate a small mismatch in optical path length between the arms of the interferometer^7^. Image acquisition and instrument control were performed with the instrument’s Vision64 software. Unlike standard optical microscopy, interferometric microscopes require the user to find the image focus and the plane where interference is present in the image. The sample must also be leveled for best results. Best practices^23,24^ were followed for all imaging in this work. Briefly, the focus was found by manually traversing the focal plan in the vertical direction. The samples were leveled using an electronically controlled tilting stage until a minimum number of interferences fringes were visible in the field of view.

### Bacterial strains and antibiotics

*Pseudomonas fluorescens* subsp. *Migula* (ATCC 12525) and *Bacillus thuringiensis* subsp. *kurstaki* HD1 (*Bacillus* Genetic Stock Center, Columbus, OH) were streaked onto lysogeny broth (LB) agar plates and incubated at room temperature for 2 days. Bacteria from isolated colonies were next grown overnight (~18 hrs) at 26 °C 225 rpm in 3 ml of LB broth.

### Bacterial agar plating and enumeration

For WLI imaging multiple 2 µl drops of bacteria in LB broth at 10^−3^ to 10^−5^ dilutions were carefully deposited onto pre-poured Trypticase™ Soy Agar (TSA) plates (BD Biosciences, 221283). For some high resolution imaging of single colonies, 2 µl drops were spotted onto self-poured LB-agar plates in which agar medium was poured to almost the top of the petri dish so that the objective could be positioned appropriately. Additionally, 200 µl of each dilution was spread-plated in quadruplicate on TSA plates so that traditional CFU spread-plating enumeration could be compared to CFU enumeration by WLI.

### Image processing and analysis

Reconstruction of 3D surface profiles from raw image data was performed by Vision64 software. Some further data pre-treatment and analysis was performed with this software such as leveling the data with planar or cylindrical form to that the agar surface was as flat as possible. CFU enumeration was conducted with Vision64’s multiple region analysis tool. Data was subsequently filtered to remove single pixel features. The tool was configured to report projected surface area, volume, and other morphological features of each colony visible in the image. The number of colonies in each imaged spot was converted to CFU/ml using a factor for the culture volume and dilution.

Open source 3D image analysis software Gwyddion^26^ (available at gwyddion.net) was used to prepare images for publication. False coloring was applied to images in a way that is consistent within each figure. As is common practice, a polynomial filter with degree 4 was applied to each image to remove waviness components and leave only surface roughness protrusions of smaller scale^24^. The goal of this image processing and filtering was to create images where the agar surface was relatively flat in order to best visualize small protrusions (i.e., those caused by bacterial colonies). Line profiles were extracted using a built-in Gwyddion tool. Images were exported in 2D and 3D forms. Images were organized and aligned into time series videos using Adobe Photoshop CS6. Data reported as means of multiple measurements have error bars showing standard deviation with the number of replicates indicated in the figure.

## Supplementary information

Supplemental Video 1

Supplemental Video 2

Supplemental Video 3

Supplemental Video 4

Supplemental Video 5

## Data Availability

The datasets generated during and/or analyzed during the current study are available from the corresponding author on reasonable request.
